# Reduction in motor error by presenting subthreshold somatosensory information during visuomotor tracking tasks

**DOI:** 10.1007/s00221-024-06887-8

**Published:** 2024-07-16

**Authors:** Toshiaki Wasaka, Shota Kano, Yoshifumi Morita

**Affiliations:** 1 Department of Electrical and Mechanical Engineering, Nagoya Institute of Technology, Gokiso, Showa, Nagoya, 4668555 Japan; 2 Center of Biomedical Physics and Information Technology, Nagoya Institute of Technology, Gokiso, Showa, Nagoya, 4668555 Japan

**Keywords:** Stochastic resonance, Manual movement, Subthreshold electrical stimulation, Grip force adjustment ability

## Abstract

Weak sensory noise acts on the nervous system and promotes sensory and motor functions. This phenomenon is called stochastic resonance and is expected to be applied for improving biological functions. This study investigated the effect of electrical stimulation on grip force adjustment ability. The coefficient of variation and absolute motor error in grip force was measured during a visuomotor tracking task under different intensities of somatosensory noise. Depending on the style of force exertion, the grip movement used in the visuomotor tracking task consisted of force generation (FG), force relaxation (FR), and constant contraction (Constant) phases. The subthreshold condition resulted in significantly lower coefficient of variation in the Constant phase and motor errors in the FG and Constant phases than the no-noise condition. However, the differences among the other conditions were insignificant. Additionally, we examined the correlation between the motor error in the condition without electrical stimulation and the change in motor error induced by subthreshold electrical stimulation. Significant negative correlations were observed in all FG, FR, and Constant phases. These results indicated that somatosensory noise had a strong effect on subjects with large motor errors and enhanced the grip force adjustment ability. By contrast, subjects with small motor errors had weak improvement in motor control. Although the effect of subthreshold noise varies depending on the individual differences, stochastic resonance is effective in improving motor control ability.

## Introduction

The fingers play a crucial role in executing daily activities involving grasping, lifting, and manipulating objects. Manual abilities, including quick or slow contraction, accurate contraction in the intended direction, and force exertion with moderate strength are essential for performing effective and purposeful dexterous movements. Impaired manual function due to cerebrovascular disease leads to difficulties in executing daily activities and this emphasizes the importance of improving finger function to maintain one’s quality of life. General approaches for enhancing motor function involve repeatedly moving and stimulating the motor areas using neuromodulation techniques, such as repetitive transcranial magnetic stimulation (Tamura et al. 2005; Kang et al. 2010) and transcranial direct current stimulation (Tanaka et al. 2009).

Stochastic resonance, a method that enhances sensorimotor function by representing sensory information noise, has gained attention. It is a phenomenon observed in various natural and artificial systems (Benzi et al. 1981) and improves the accuracy and efficiency of sensorimotor functions through sensory noise in biological systems (Douglass et al. 1993; Collins et al. 1996). In humans, the sensory information of electrical stimuli below the sensory threshold of the vestibular system reduces postural sway (Pavlik et al. 1999; Fujimoto et al. 2016), and mechanical stimuli improve balance control (Priplata et al. 2006) and manual dexterity (Seo et al. 2014; Nobusako et al. 2019). However, as the effects of stochastic resonance on the motor system mostly focus on the control of complex movements, how it affects the control of muscle contraction through the presentation of sensory noise has not yet been elucidated. Thus, this study aimed to examine whether adding electrical noise reduces motor errors and investigate how stochastic resonance influences grip force adjustment ability in various muscle contraction types.

Somatosensory information is used to monitor the recognition of objects in contact with the skin, joint movement, and force exertion. Previous studies have shown that the activity of Ia fibers from muscle differs depending on the type of muscle contraction (Burke et al. 1978; Hulliger and Vallbo 1979; Al-falahe et al., 1990). The effects of weak somatosensory noise may differ depending on the type of muscle contraction. Therefore, to control the muscle contraction style, we adopted a visual tracking task in which force is slowly adjusted. The pattern of muscle force development can be categorized into three styles: force generation (FG), in which the muscle force gradually increases; force relaxation (FR), in which the force gradually decreases; and constant force exertion (Constant). Subthreshold electrical stimulation of the tibial nerve during constant isometric contraction of plantar flexion reduces the absolute error from the target force value (Kouzaki et al. 2012). However, whether the noise from electrical stimulation has a similar effect on different contraction types remains unclear. Our previous results showed that the amplitude of somatosensory evoked potentials was suppressed during movement and differed between force generation and relaxation phase of grip movement (Wasaka et al. 2012). Since information processing of the somatosensory system differs depending on the type of force exertion, it is assumed that the influence on the motor system also differs; hence, we examined electrical stimulation slightly stronger than the sensory threshold. Using a visuomotor tracking task with three styles of force exertion, we investigated the effects of somatosensory noise on the ability to adjust muscle contraction.

## Materials and methods

### Participants

The participants in this study were 16 healthy students with an average age of 22.6 ± 1.1 years, consisting of 14 men and 2 women. They were right-handed, as assessed by the Edinburgh Handedness Inventory.

### Motor task

This study employed a visuomotor tracking task using a device that quantitatively evaluates grip force adjustment (iWakka, I’m Co. Ltd., Aichi, Japan). When force is applied to iWakka with fingers, the plate spring deforms and changes its width. A strain gauge attached to the plate spring measures, amplifies, and incorporates this change into a computer in real time to visualize the changes in grip force. When the participants grasped and increased their grip force, a leaf spring inside the measurement device, iWakka, deformed, causing the outer diameter to decrease. The relationship between the deformation and the grip force is linear, with a force exertion of 0.483 N resulting in a deformation of 1 mm.

The participants were instructed to deeply sit in a chair during the measurement with their backs straight and their lower backs barely touching the backrest. Both legs were opened to approximately shoulder width, and the knee joint was bent to approximately 90°. Their feet were kept on the ground during the measurements. The right elbow was placed on the chair armrest to ensure that the weight of the upper body was not felt, and all five fingers gripped the iWakka. During the motor task, the iWakka was not lifted but was placed on the desk.

**Fig. 1:**
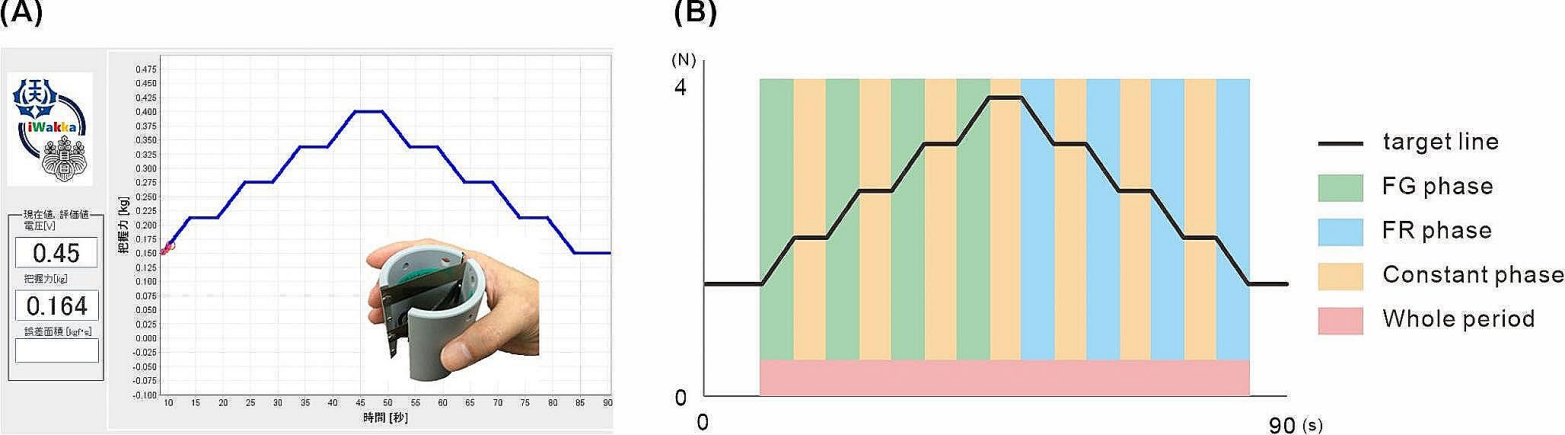
(**A**) Device for measuring grip force and measurement screen. (**B**) Target force line of visuomotor tracking task. The target force line was set as a “mountain” shape, and the participants adjusted their grip force to match the target line. There is a red pointer that indicates the grip force in at the center of the laptop screen, and when the subject increased the force, the pointer moved upward. The target force line flows from right to left. This motor task consisted of three force exertion modes: the force generation phase (FG phase), the force relaxation phase (FR phase), and the constant contraction phase (Constant phase)

Figure 1 shows the target force line of visuomotor tracking task and phases depending on the types of muscle force exertion. The participants were instructed to accurately match the target force displayed on a computer monitor using their right hand, with visual feedback regarding the force exerted on the same monitor (Kaneno et al. 2017). Initially, participants were instructed to focus on a central point on the monitor that moved up and down according to the level of force exertion. The target force line was set as a “mountain” shape, ranging between minimum and maximum values of 1.471 N and 3.923 N, respectively. The participants adjusted their grip force to 1.471 N at the beginning of the motor task. The target force was increased by 0.613 N over 5 s at a consistent pace and remained at this new level (2.084 N) for 5 s before increasing again by 0.613 N to a new plateau (2.697 N). This process was repeated twice until the maximum value was reached. The reverse sequence was then presented until the target force returned to 1.471 N. The duration of each trial was 90 s.

The visuomotor tracking task consisted of three force exertion modes: the FG phase, in which the participants exerted muscle force while moving their fingers in the direction of closing the iWakka; the FR phase, in which the participants exerted muscle force while moving their fingers in the direction of opening the iWakka; and the Constant phase, in which the participants exerted constant muscle force without change.

### Electrical stimulation

The right median nerve, which innervates the thumb and index and middle fingers, was stimulated at the wrist using a saddle-type electrode (NM-422B, Nihon Kohden). The square-wave pulse waveform of electrical stimuli (0.2 ms duration), used as noise for somatosensory information, was randomly presented at inter-stimulus intervals of 20, 40, 60, 80, and 100 ms using Psychopy software. Two conditions were set for the intensity of electrical stimulation based on the sensory threshold. To determine the sensory threshold, the stimulus intensity was confirmed by gradually increasing from an unperceivable intensity, and then gradually decreasing it from a perceivable intensity. This procedure was repeated twice. In addition, we measured the motor threshold. Since we did not record an electromyogram, we carefully observed the movements of the thumb visually and confirmed the intensity at which small twitch occurred. As stochastic resonance studies generally use subthreshold somatosensory information, we set the intensity of the sensory threshold to 0.9 times (ST0.9 condition) its intensity and a slightly higher intensity of 1.1 times (ST1.1 condition). Additionally, measurements were performed under a control condition without electrical stimulation (NO condition).

### Measurement of grip force adjustment ability with electrical stimulation

The participants practiced the same visuomotor tracking task three times before measurement for familiarization. In each condition, three trials were conducted with a break of approximately 1 min between the trials, and the order of the three conditions was randomized with a 3 min break. The participants were not informed which of the three conditions was presented during the motor task. After all measurements, the participants were informed the order of stimulus conditions and verbally asked whether they were able to detect the stimulus under the ST0.9 condition below the sensory threshold. The grip force adjustment ability was evaluated to calculate the coefficient of variation and absolute error between the actual grip force and target force every 0.1 s.

**Fig. 2:**
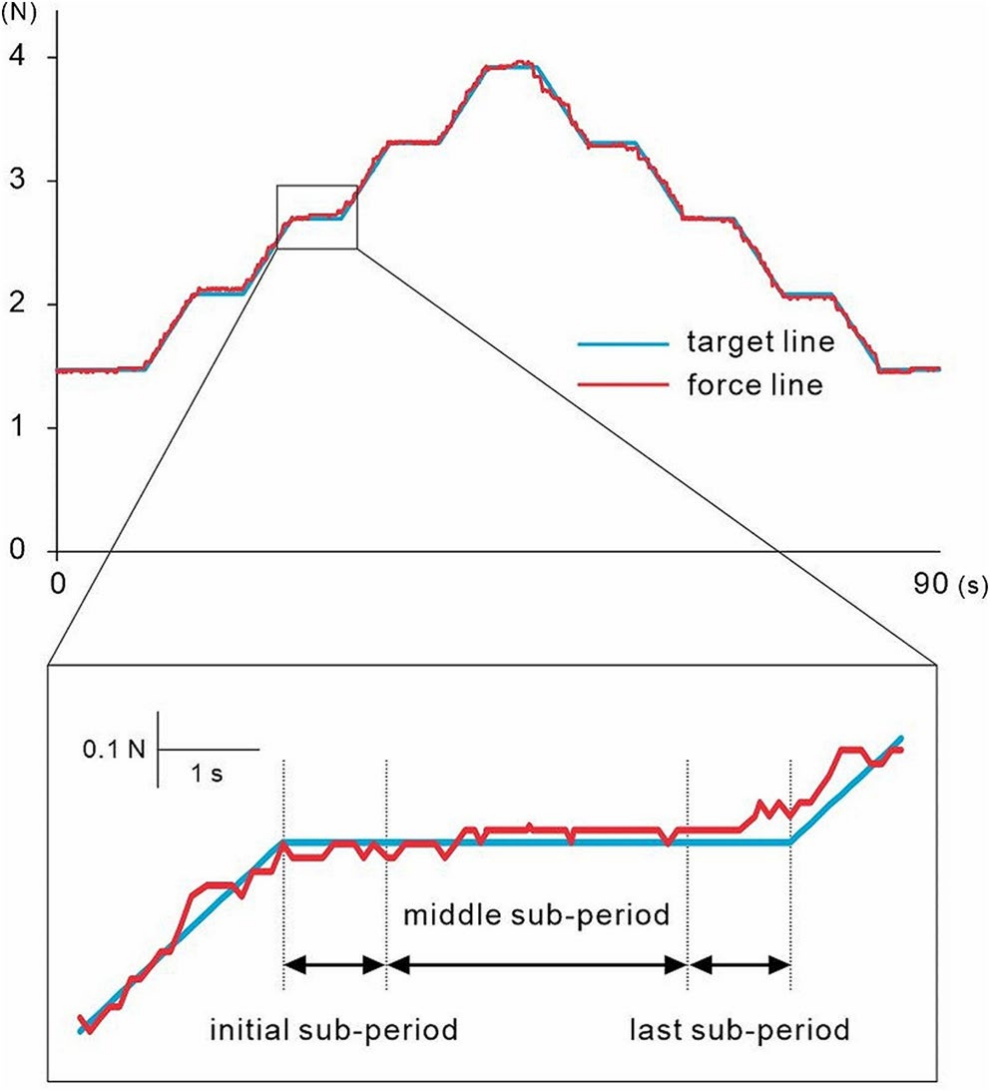
Result of one trial from one representative subject. The light blue and red lines indicate the target and force lines, respectively. A portion of the trial is enlarged at the bottom. The first 1 s of each phase is a sub-period of instability due to switching the type of force exertion (the first sub-period), the middle 3 s is a sub-period of stable movement (the middle sub-period), and the last 1 s is a sub-period of continuing movement while preparing for the next movement (the last sub-period)

The visuomotor tracking task changed the muscle contraction style every 5 s. Therefore, we analyzed each phase by dividing it into three sub-periods (Fig. 2). The first 1 s of each phase was a sub-period of instability due to switching the type of force exertion (the first sub-period), the middle 3 s was a sub-period of stable movement (the middle sub-period), and the last 1 s was a sub-period of continuing movement while preparing for the next movement (the last sub-period).

### Statistical analysis

The coefficient of variation and absolute error under the three stimulus conditions was compared over the three sub-periods of each phase by using the Friedman test, which is a non-parametric repeated-measures of variance (ANOVA), to determine whether noise affected grip force adjustment ability. The three stimulus conditions were compared for the first 1 s, middle 3 s, and last 1 s of the FG, FR, and Constant phases. When a statistically significant difference was observed in ANOVA, post-hoc analyses were conducted using the Wilcoxon signed-rank test with Bonferroni correction. Statistical significance was set at *p* < 0.05.

Subsequently, we investigated the correlation between the motor error in the NO condition and its change with subthreshold electrical stimulation in 16 subjects and performed linear regression analysis (Pearson correlation). Statistical significance was set at *p* < 0.05.

## Results

### Stochastic resonance effect of electrical stimulation on grip force adjustment ability

The mean intensities of sensory and motor thresholds were 5.900 ± 1.889 mA and 11.625 ± 2.130 mA, respectively. When questioned after the measurement, all participants reported perceiving very weak stimuli in the ST1.1 condition, while they were unable to detect any stimulus in the NO and ST0.9 conditions. Table 1 shows the coefficient of variation in the three sub-periods of the FG, FR, and Constant phases. ANOVA revealed a significant difference in the middle and last sub-periods of the Constant phase (*p* < 0.05, both), no difference was observed in any sub-period of the other phases. Multiple comparisons showed that the coefficient of variation was significantly smaller in the ST0.9 condition than in the NO condition in the middle and last sub-periods (*p* < 0.05, both).

**Table 1:** Coefficient of variation in the three sub-periods of FG, FR, and constant phases

Style		Initial (1 s)				Middle (3 s)				Last (1 s)		
		NO	ST0.9	ST1.1		NO	ST0.9	ST1.1		NO	ST0.9	ST1.1
FG	Mean	1.677	1.634	1.802		4.268	4.161	4.21		1.389	1.100	1.115
	SD	0.504	0.364	0.484		0.421	0.337	0.302		0.523	0.337	0.318
FR	Mean	1.329	1.367	1.367		4.369	4.347	4.266		1.524	1.483	1.694
	SD	0.346	0.291	0.278		0.32	0.304	0.263		0.360	0.381	0.397
Constant	Mean	0.703	0.607	0.681		0.731	0.569*	0.653		0.673	0.426*	0.497
	SD	0.383	0.242	0.310		0.225	0.176	0.225		0.282	0.131	0.222

*P < 0.05 (between the NO and ST0.9 conditions)

FG, force generation; FR, force relaxation; Constant, constant contraction

**Fig. 3:**
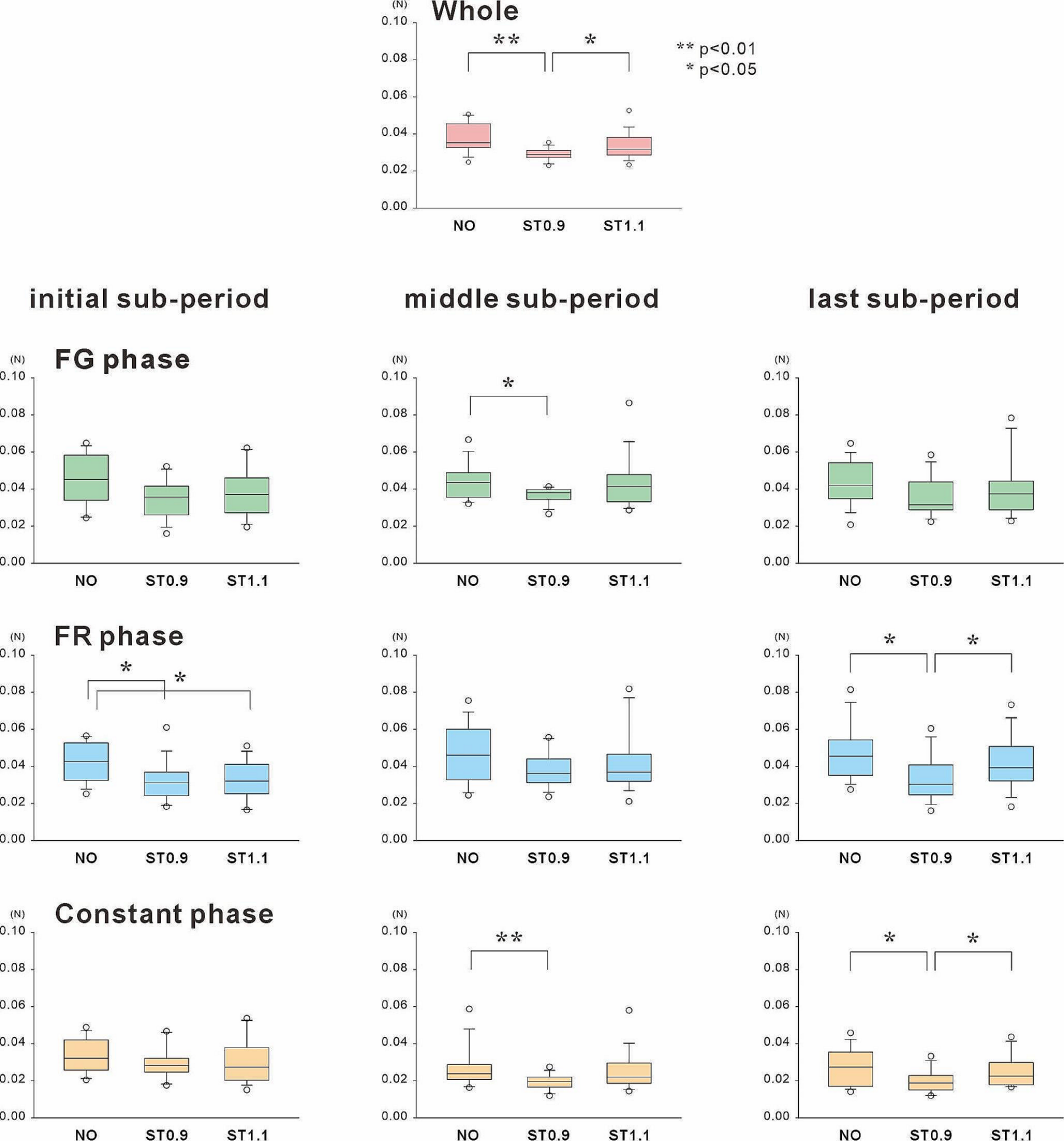
Motor errors (*n* = 16) of the visuomotor tracking task in the whole period and three phases of force exertion styles. The visuomotor tracking task consisted of three force exertion modes: force generation phase (FG phase), force relaxation phase (FR phase), and constant contraction phase (Constant phase). The motor error was significantly smaller in the ST0.9 than in the NO and ST1.1 conditions during the entire period of the visuomotor tracking task. The stochastic resonance effects were not similar when examining the differences in the force exertion styles. In the middle sub-period, a significant reduction in motor error was observed in the FG and Constant phases in the ST0.9 condition compared with those in the NO condition. In contrast, reduction of motor errors in the FR phase was observed in the initial and last sub-periods

The absolute error between the target value and the grip force over the entire visuomotor tracking task period was 0.037 ± 0.008 N, 0.029 ± 0.003 N, and 0.033 ± 0.007 N, and in the NO, ST0.9, and ST1.1 conditions, respectively (Fig. 3). ANOVA revealed a significant difference among the three conditions (*p* < 0.01). Multiple comparisons showed that the motor error was significantly smaller in the ST0.9 condition than in the NO (*p* < 0.01) and ST1.1 conditions (*p* < 0.05).

### Change of motor error in the sub-periods of each contraction style

We investigated whether somatosensory information with different intensities modulated the ability of grip force adjustment in each contraction pattern because the visuomotor tracking task involved three different modes of muscle force exertion, the FG, FR, and Constant phases (Fig. 3).

In the initial sub-period, ANOVA using the difference in stimulus condition as a factor revealed a significant difference only in the FR phase (*p* < 0.05). Post-hoc analysis showed that absolute motor errors in the ST0.9 condition were significantly smaller than those in the NO and ST1.1 conditions (*p* < 0.05, both).

In the middle sub-period, the results of ANOVA showed a significant difference in all contraction styles (*p* < 0.05, all). In multiple comparisons, absolute errors in the ST0.9 condition were significantly lower in the FG (*p* < 0.05) and Constant phases (*p* < 0.01) than in the NO condition. The differences under the other conditions were insignificant.

In the last sub-period, ANOVA showed a significant difference in the FR and Constant phases (*p* < 0.01, both). In both phases, absolute motor errors were significantly lower in the ST0.9 condition than in the NO and ST1.1 conditions (*p* < 0.05, in all comparison).

### Change of motor error by stochastic resonance effect

**Fig. 4:**
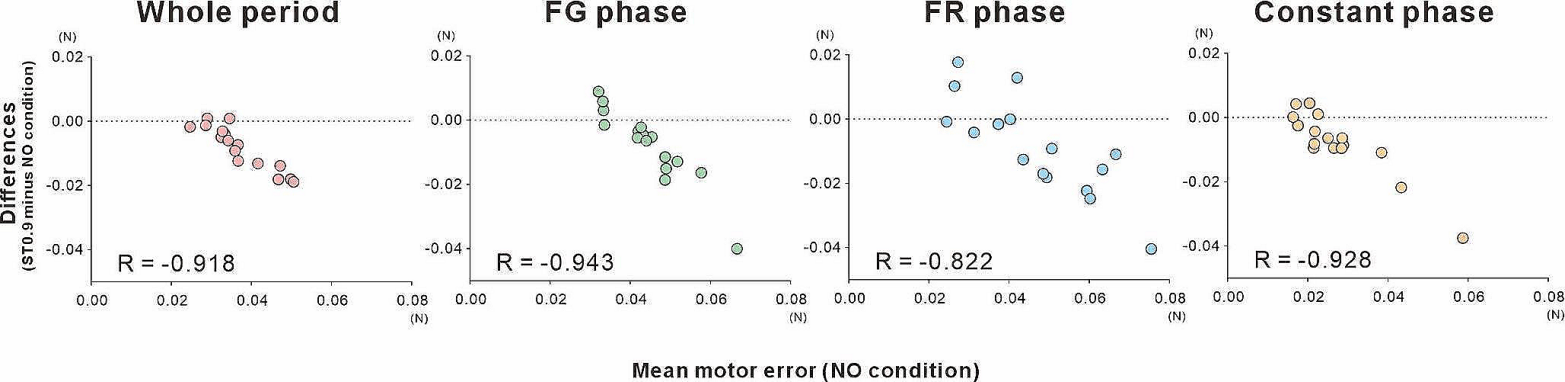
Correlation between the motor error in the NO condition and the change in motor error induced by subthreshold electrical stimulation in all participants. In the whole period and the three phases of middle sub-period, there has been a gradual decrease with subthreshold electrical stimulation depending on the amount of motor errors in the NO condition

Figure 4 shows a scatter plot of the change on motor error induced by subthreshold somatosensory stimulation. In the whole period and three phases, the motor error during weak electrical stimulation exhibited an almost linear decrease with increasing motor error in the NO condition. Statistical analysis showed a significant negative correlation (whole period: *R* = -0.918; FG phase: *R* = -0.943; FR phase: *R* = -0.822; Constant phase: *R* = -0.928; *p* < 0.01) between the motor error in the NO condition and the changes induced by weak electrical stimulation below the sensory threshold in the ST0.9 condition.

## Discussion

The presentation of weak noise effectively enhances the detection of stimuli in the sensory systems and perceptual processes (Collins et al. 1996, 1997; Wells et al. 2005). Our results showed that subthreshold somatosensory information obtained using electrical stimulation reduced the motor error from the target value in the visuomotor tracking task. Subthreshold electrical noise induced stochastic resonance and improved grip accuracy, but substantial inter-individual differences were observed. Notably, participants who exhibited large motor errors during the visuomotor tracking task were considerably affected by somatosensory noise in the sensorimotor system.

The sensory system processes information from inside and outside the body, providing information for motor control via the central nervous system. Stochastic resonance has been hypothesized to affect the motor system by facilitating the sensory system. Electrical stimulation of the vestibular system has been used to improve postural balance in the elderly (Fujimoto et al. 2016). In the somatosensory system, the presentation of subthreshold somatosensory information improves the ability to move pegs and square boxes, as well as the maximum precision grip force in patients with upper limb motor dysfunction (Seo et al. 2014; Nobusako et al. 2019). However, improvements in motor function due to somatosensory noise have been reported in the performance of all motor tasks, and how the stochastic resonance effect extends to various types of muscle movements is unclear. We investigated whether somatosensory information improves motor accuracy by using a visuomotor tracking task and demonstrated that the noise reduces motor errors in various sub-periods in the FG, FR and Constant phases.

Subthreshold electrical stimulation was previously shown to reduce force fluctuations during low-level, steady force exertion of the lower muscles (Kouzaki et al. 2012). Our study using grip movement revealed the same result, with the coefficient of variation being considerably small during the Constant phase in the ST0.9 condition. Additionally, although our study demonstrated a significant reduction in the absolute motor error during the FG phase, the difference in the FR phase was insignificant. These results indicate that subthreshold electrical stimulation enhanced the ability to slowly increase grip force but not to slowly decrease it; however, the stochastic resonance effect varied across different muscle contraction modalities. Afferent activity from muscle spindles differ depending on the contraction style (Burke et al. 1978; Al-falahe et al., 1990). In addition, a neuroimaging study comparing FG and FR in humans showed lower activation of the primary motor cortex during force relaxation (Spraker et al. 2009). Similarly, somatosensory information processing in the primary somatosensory cortex differed between FG and FR (Wasaka et al. 2012). These findings indicate that the activity of the cortical sensorimotor areas differs depending on the muscle contraction pattern. In the present study, the intensity of the electrical stimulus presented during the visuomotor tracking task was constant, but stochastic resonance by sensory noise seemed to have different effects depending on the muscle contraction modes.

Noise enhances sensorimotor integration processes involved in motor regulation. Since somatosensory information plays a crucial role in motor accuracy and force regulation (Rothwell et al. 1982), it is conceivable that the facilitation of the somatosensory system by stochastic resonance affects motor control ability. Variability in the discharge rates of motor units and the relationship between agonist and antagonist activities causes motor errors (Vallbo and Wessberg 1993; Laidlaw et al. 2000). Our finding that noise reduces motor errors suggests that somatosensory information slightly weaker than the sensory threshold affects these underlying mechanisms. In the present study, electrical stimulation was delivered to the median nerve that innervates the first lumbrical, second lumbrical, opponens pollicis, flexor pollicis brevis and abductor pollicis brevis muscles. Therefore, this stimulation is assumed to affect sensorimotor integration between the somatosensory and motor systems in the finger muscles innervated by the median nerve. In the tracking task similar to our motor task, the alpha-gamma linkage increases the afferent discharges from muscle spindle (Hulliger and Vallbo 1979). The possible explanation for reduction of motor errors might be that subthreshold electrical stimulation caused excitation in Ia fibers from the muscle spindle, inducing stochastic resonance and affecting the alpha-gamma linkage. Another possible explanation of our finding is that electrical stimulation caused excitation of Ib fibers from the Golgi tendon organs or cutaneous information acted on the motor system (Cordo et al. 1996; Collins et al. 2005). At intensities below or just above the sensory threshold, the cutaneous nerves appear to be excited, but because Ia and Ib fibres have similar diameters, it is unclear whether electrical stimulation produces differences in their activation. Therefore, the region of the central nervous system involved in promoting sensorimotor function in response to noise has not been clarified. During a tactile discrimination task using fingertips, discrimination ability is improved when noise below the sensory threshold is presented to the fingertips; however, discrimination ability is improved even when noise is presented to the dorsum of the hand or thumb, which is not related to discrimination (Lakshminarayanan et al. 2015). Although our results showed that presenting noise to the nerve innervating the moving muscles improved motor performance, further investigation is required to elucidate the effects of presenting tactile information to other body parts related to the movement.

Somatosensory information is transmitted to the spinal cord and cerebral cortex. The stimulus intensity of the sensory threshold evoked neural activity in the primary somatosensory cortex (Lin et al. 2003), and presenting continuous noise of somatosensory information below the sensory threshold enhanced the amplitude of somatosensory-evoked potentials (Seo et al. 2015). Additionally, when stochastic resonance by sensory noise improves motor accuracy, corticomuscular coherence increases (Trenado et al. 2014). These findings suggest that feedback of somatosensory information from the muscles and skin exists owing to grip movement, and a neural mechanism that reduces motor errors by noise possibly exists in the sensorimotor system.

As electrical stimulation is applied to the wrist, the influence of attention on tactile information is assumed to improve the ability to adjust the grip force of the fingers. However, improvement in the grip force adjustment control was more pronounced with the subthreshold intensity of the ST0.9 condition than that of the NO condition. Under these conditions, the participants were unable to judge whether electrical stimulation was being presented during the motor task. Thus, factors other than the influence of attention on electrical stimulation acting on the sensorimotor system improved the grip force adjustment ability.

Previous studies investigating motor performance using stochastic resonance have shown that subthreshold somatosensory noise is effective (Kouzaki et al. 2012; Mendez-Balbuena et al. 2012; Germer et al. 2019); however, the intensity above the sensory threshold has not been investigated. Since the ST1.1 condition did not considerably improve motor performance, our results suggest that a higher intensity of noise does not enhance motor adjustment ability. Because the median nerve contains both motor and sensory nerves, stronger stimulation intensity increases sensory nerve activity and simultaneously excites motor fibers. Motor nerve activity may have caused recurrent inhibition in motor neurons (Hultborn et al. 1971), and motor errors may not have changed in the ST1.1 condition. Given that the mean sensory threshold in all subjects was 50.7% of the motor threshold, the intensity in the ST1.1 condition induced no excitation of motor fibers, thereby exerting minimal influence on recurrent inhibition. Although we found that stimulus intensity lower than the sensory threshold is important to induce stochastic resonance for enhancing motor ability, it remains unclear whether this intensity is optimal.

A significant correlation indicated that the effect of weak electrical stimulation was more for participants with larger absolute motor errors in the NO condition across all contraction phases. However, no significant differences in absolute motor errors were observed between the NO and ST0.9 conditions in the FR phase, although it decreased in the ST0.9 condition. Based on these results, we suggest the discrepancy between results of ANOVA and correlation analysis is attribute to the large variations in the amount of reduction during the FR phase. In addition, the correlation analysis showed that weak electrical stimulation had a large stochastic resonance effect on the participants with large motor errors in the NO condition but had little effect on those with small motor errors. Establishing a method to enhance the motor function of those with small motor errors in the NO condition is important. Further studies to resolve the issue of individual differences by improving the noise presentation method are warranted.

Appropriate sensory information processing and sensorimotor integration in the central nervous system enables the coordination of motor control, resulting in postural maintenance, motor accuracy, and force coordination. We found that the weak sensory information reduced motor errors in various styles of muscle contraction. In addition, this phenomenon showed characteristic changes in the sub-periods before and after changing the contraction styles and when performing the same contraction style stably and continuously. Our research is an experimental verification in a limited setting involving switching between muscle contraction types and continuing a stable contraction. Although it is unclear from our results whether the subthreshold sensory noise influenced on grip force control based on the visual information of exerted force or the ability to accurately control muscle position, our findings demonstrate that stochastic resonance can be applied for precise motor control in humans. Given that our research results are expected to be applied to improving daily movements using fingers as well as specialized motor skills, they have important applications in improving rehabilitation and the development of physical abilities.

## Data Availability

The data are available from the corresponding author upon reasonable request.
